# Pathways to reimagining commercial health insurance in India

**DOI:** 10.3389/fpubh.2022.1006483

**Published:** 2022-11-23

**Authors:** Hasna Ashraf, Indradeep Ghosh, Nishanth Kumar, Anjali Nambiar, Sowmini Prasad

**Affiliations:** Dvara Research, Chennai, Tamil Nadu, India

**Keywords:** commercial health insurance, insurance demand, insurance supply, information asymmetries, health outcomes, financial protection, integration

## Abstract

In this paper we explore how India's growing commercial health insurance (CHI) segment can be reformed to deliver adequate financial protection and good health outcomes. We lay out key issues in the demand- and supply-sides of the insurance market that need to be addressed for CHI to be more aligned toward universal health coverage (UHC). On the demand side, we identify a consumer who strays far from the rational actor paradigm and therefore one whose needs require a fundamentally different approach than the one that commercial health insurance in India has so far taken. We lay out precisely the different stages involved in bringing a consumer to the insurance market and the conditions under which that consumer is likely to purchase insurance. On the supply side, we describe the many concerns that a new entrant into the commercial health insurance market must grapple with. We conclude with a set of pathways that brings the two sides of the market together to shed light on possible pathways for reform in the commercial health insurance sector in India. Despite the many challenges that this sector faces in India, we believe that there is room for optimism, and with the right amount of regulatory foresight, even room for radical transformation.

## Introduction

Over the past decades, India has made considerable progress in citizen health outcomes, as evidenced by significant improvements in standard indicators such as infant and maternal mortality. However, the disease burden in India continues to be disproportionately high, and malnutrition and other risk factors for disease and injury are widespread. With a DALY of more than 33,000, India's health outcomes are much poorer than those of her neighbors, Sri Lanka (DALY 26,178) and Bangladesh (DALY 27,077) (1). India is currently experiencing a “double whammy” of diseases, with rising non-communicable diseases (NCDs) adding to the already existing burden of high maternal and child health concerns. A highly fragmented provider landscape (2) and the resultant reinforcement of poor consumer behavior (e.g., doctor shopping) does little to improve health outcomes. The rising cost of healthcare exacerbates the problem further. With more than 55% of the total health spending coming Out-of-Pocket (OOP) (3), increasing health costs add significantly to the financial burden of households, causing about 7% of the population to fall into poverty annually (4). In the context of poor health outcomes and the rising financial burden on households caused by OOP expenditures, there is a need to innovate alternate financing strategies to rectify the situation.

Though relatively nascent, India has a thriving commercial health insurance industry, growing at the rate of more than 20% annually. This segment which came into existence with the opening up of the insurance market in 2000, currently caters to 136.7 million (~10% of the population) through voluntary group and individual businesses and 362 million (~26.5% of the population) through government business (5). By commercial insurance, we refer to private players and public sector companies like New India Assurance, United India Insurance, etc., who commercially offer health insurance. In addition, this space is currently occupied by both general and stand-alone health insurers, with hospitalization-based indemnity contracts being the primary products offered.

In this paper, we will explore pathways for commercial health insurance (CHI) to deliver adequate financial protection as well as good health outcomes. First, in the next section, we will define the problem that our paper seeks to analyse (but not solve), and draw out its connection to two related problems, the problem of securing universal health coverage (UHC) and the problem of reducing and/or optimizing OOP expenditures. Then, in Section Breaking down assumptions: Following the consumer's journey, we take up the demand-side of the CHI market and explore the issues there. In Section Determining supply, we take up the supply-side and perform a similar exercise. In section Getting the right alignment of incentives, we highlight the problem information asymmetries cause both insurance demand and supply. Finally, in Section Discussion: In search of a solution, we outline broad solution pathways for reform.

## The problem of rethinking CHI

We begin with the observation that most CHI contracts in India take the form of indemnity insurance for hospital stays and/or visits. Do such insurance contracts make a good deal of sense? At first glance, it would appear so. In a world where the demand-side of the insurance market is populated by rational actors who are endowed with just the right amount of self-knowledge about their health concerns as well as their risk-bearing capacities (i.e., who know their utility functions defined over health outcomes as well as those defined over lotteries), the optimal design of insurance would indeed call for indemnity contracts. These contracts would cover low-frequency high-impact health shocks, where the impact is high in the sense of imposing a significant financial burden on an uninsured consumer for what is sometimes called tertiary care. As long as the insurance company can amass a sufficiently large number of consumers to sell such contracts to, the laws of statistics guarantee a commercially viable operation and make the supply of insurance feasible at a price that consumers, having performed their own risk-calculations, will be willing to pay (6, 7). In such a world, insurance contracts will *not* cover high-frequency, low-impact health “shocks” such as primary or preventive care check-ups. So it is necessary to write into such contracts a suitable deductible or co-pay element.

CHI contracts in India appear to satisfy the indemnity aspect of the above design principles, but not others. For one, coverage caps are quite low, so these contracts do not appear to incorporate the logic of insurance needing to cover high-impact health shocks. Further, there is typically no deductible.

When the theoretical setup of a rational consumer is overlaid with the presence of information asymmetries between the two sides of the market, we arrive at the canonical model that the theoretical literature on health insurance contracts has attempted to solve. In this model, the optimal design of insurance must now wrestle with adverse selection and moral hazard problems.

Notwithstanding these objections to the form of CHI contracts in India, the point we wish to make is broader and deeper. This is that the canonical model is not the correct approach for thinking about insurance market design, and this is because the rational actor assumption is very difficult to validate in the healthcare domain. Firstly, one's personal health is a matter about which one may have little information without any prior medical intervention, but there is a range of behavioral factors that impede the very seeking of such knowledge (about one's own health condition). Secondly, even when such knowledge becomes available, the tendency to doubt the doctor's diagnosis, or to shop around for a second or third opinion, is itself a behavioral adaptation to the highly complex and multifaceted nature of the “good” or “commodity” that is in play – one's health condition and its tending to – and the context in which such knowledge-seeking occurs – typically, a highly fragmented supply-side, as already described in the introduction. Thirdly, even if the doctor's diagnosis is trusted, the capacity to recognize that the health condition is best addressed *via* a combination of preventive care and insurance, cannot be taken for granted on the part of the consumer. Indeed, the best possible supply-side response to the non-rational healthcare consumer is itself a contested subject.

The above points about actual consumer characteristics compel us to recognize that a good CHI market/system should serve a two-fold objective: good health outcomes *and* adequate financial protection. This is the problem that we seek to analyse in this paper. The objective is dual because the tending to one's health and the capacity to pay for such tending cannot be separated. Indeed, many of the behavioral factors that impede a rational outlook toward purchasing health insurance are the same factors that impede a rational outlook toward caring for one's own health. This is also the reason that OOP expenditures tend to be inefficient in a country like India (i.e., high without the consequent benefits of good health outcomes). The problem of optimizing OOP expenditures may therefore be linked to the problem of designing suitable CHI contracts that serve the dual objective. In turn, the statement of a dual objective renders obvious the need to think about the supply-side of the CHI market from the perspective of integrating the provision and financing functions. This is, therefore, the approach we take in constructing our hypotheses.

Since the dual objective arises naturally in the context of actual consumer characteristics, as opposed to the context of an idealized rational actor who is endowed with a very high degree of self-knowledge, it stands to reason that any system for UHC must also adopt such a dual objective. Yet, public health systems in many developing countries, and certainly in India, do not appear to explicitly incorporate such an objective even if they embed the integration of provision and financing. For example, in India, the PMJAY scheme integrates non-contributory financing with public sector provision but not in a way that encourages better health outcomes. This then raises the question of whether the integration of contributory financing schemes with provision is a necessary condition for delivering on the dual objective. This is not a question we take up in this paper. However, we note that some scholars have argued for doing away with tax-funded health insurance altogether and replacing it with voluntary health insurance, in which the problem of affordability of insurance is solved with suitable public subsidies (8). The relevant implication for such an argument is that CHI could then be deemed a pathway to UHC. The wrinkle that would remain to be ironed out is whether a vision for UHC is consistent with a vision for voluntary insurance – perhaps not, if behavioral factors prevent people from taking up insurance even when they have understood its benefits. In such a case, UHC would require the mandating of insurance purchase by every citizen, and CHI would become the *only* pathway for UHC with public subsidies being used to solve the affordability problem for sections of society that would otherwise be excluded. We do not suggest this extreme possibility as a desirable normative outcome, but rather only to facilitate an appreciation of some of the issues linking CHI to UHC.

## Breaking down assumptions: Following the consumer's journey

**Figure 1:**
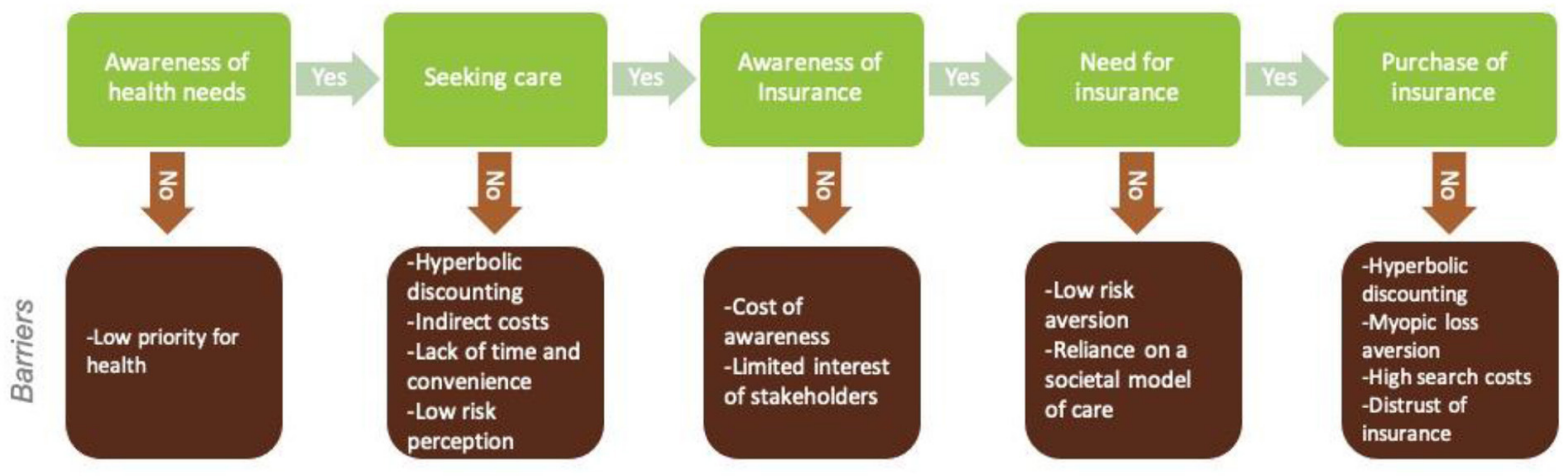
Decision mapping.

To understand factors affecting the demand for insurance, it is necessary to trace the individual's decision pathway and the contexts in which they take decisions. Figure 1 models this decision pathway of insurance demand.

### Awareness of health needs

To demand insurance, individuals must be aware of and be willing to acknowledge their health needs to begin with. While this may seem simple enough, such awareness and acknowledgment of health needs are largely limited. Adequate knowledge regarding even routine breastfeeding practices, for example, was found in only one-third of antenatal mothers studied (9, 10). In a study conducted to identify factors associated with delay in seeking care for tuberculosis in South India, nearly 40% of those who delayed seeking care attributed it to lack of awareness (11). A study conducted among adolescents in Uttarakhand found awareness of various health issues to be low; only 12% in rural areas and 48% in urban areas were aware of anemia (12). Low priority to health could be one reason for this low awareness and acknowledgment of health needs (13).

### Seeking care

Even when an individual is aware of his/her health needs, it may not be enough to get him/her to seek care. One reason for this could be that individuals do not think in terms of their long-term interests. As a result, they may tend to bias the present (*present bias*) and place less weight on future payoffs as this future becomes more distant (*hyperbolic discounting*) (14, 15). This could imply that, even when they know the benefits of seeking care, when it comes to actually seeking care, individuals may see less value for it in the present and try to delay it.

The indirect costs involved in seeking care, in addition to the direct cost of healthcare, could be yet another reason for individuals to hesitate to seek care. A study conducted in 2012 in India found that in rural areas, only 37% of people could access in-patient facilities within a 5 km distance, and 68% were able to access out-patient facilities. Here, indirect costs in the form of transportation and loss of earnings resulting from travel time led to delays in care-seeking (16). Even when care is accessible, the effectiveness of this access may be limited due to insufficient infrastructure (16) competent providers (17, 18). Other reasons for delayed care-seeking include lack of time and convenience and low-risk perception (19).

### Awareness of insurance

The next barrier to insurance demand is the lack of knowledge regarding insurance itself. Public awareness of health insurance in India is poor (20, 21) and this poor understanding of insurance is not limited to the poor and illiterate; even India's educated middle class has trouble in understanding insurance (20). Additionally, there exists confusion in the minds of individuals between different types of insurance and investment products (22). Given the geographical dispersion and illiteracy, increasing awareness, particularly among the rural poor, is quite costly. Insurance agents have not viewed these households as an attractive market and, as such, have taken little to no effort to educate them (23).

### Need for insurance

Even when individuals are aware of insurance, they do not necessarily find the need to buy one. Low-risk aversion of individuals could be one possible reason. This can be traced back to some historical and cultural factors associated with traditions of care and financial management, which are carried out at the household level by extended family. This societal model of caring in India acts as implicit insurance. It has led to a reduced acceptance of risk and a loss of the sense of individual responsibility to cover for risk proactively. Essentially, it is as if individuals have left risks to society and destiny and, as such, may actively feel little need for insurance even when they have a basic awareness of insurance products (23).

### Purchase of insurance

Finally, even when individuals feel the need to buy insurance, a number of factors prevent the actual purchase. As in the case of care-seeking, a bias toward the present and discounting of future costs and benefits may be at play here. Future costs and benefits are so heavily discounted that even a small investment at present seems excessive. Another reason underlying this reluctance to purchase insurance could be the tendency to assess risks in isolation and treat losses as more painful than the pleasure from gains (*myopic loss aversion*) (7). This pain and pleasure are understood with the current level of wealth in mind. Individuals may fail to see what can potentially happen to this wealth in the future in the absence of risk protection. Additionally, the idea of paying premiums and getting nothing back is one that individuals may find difficult to comprehend (8, 24).

Insurance products in India are quite complex to understand and compare and would require significant effort on the part of the individual to identify a suitable product. If these search costs are high compared to the expected utility that they hope to gain from the search, individuals may refuse to put in the effort and choose to remain uninsured (7). Additionally, the channel costs associated with India's indemnity products is quite high, making the product much more expensive that the risk reduction benefit is not adequate.

Yet another factor that prevents purchase is the lack of trust in insurance. This could be higher when there has been a history of default. The lack of trust in insurance is reinforced when the claims process is difficult, and the payout of claims is either delayed or, even worse, denied (8, 23). This may also be an inherent limitation of the indemnity model, which addresses such complex problems on an arms-length basis. India's current stand-alone health insurance market with a claims ratio of 64% (5) does little to build consumer confidence in the market. Trust can be seen as an intertemporal reputation problem involving the larger insurance ecosystem with different stakeholders (insurers, providers, etc.).

## Determining supply

The supply-side of the healthcare insurance market is inextricably intertwined with the supply-side of the healthcare provider market, and this produces both a confluence and a conflict of interests that need to be carefully thought through. Additionally, there is the matter of how the supply- and demand-side interact, and the information asymmetries intrinsic to those interactions. The complexity of the issues that therefore arise can perhaps be addressed using a stage-by-stage approach [see (25) for one such approach], as we have done for the demand-side, but here, we take the simpler, albeit messier, route in laying out some of the broad features of the supply-side problem, leaving their proper organization into a more rational framework to future work.

### Entry decision

We begin by asking why a capitalist entrepreneur will want to enter the health insurance market at all. The answer must be that he/she finds it profitable (at the margin) to do so. What, therefore, are the conditions of profitability in the insurance market? At the very least, the opportunity to pool risks must exist, and this opportunity must exist over a large customer base, because only then will the law of large numbers guarantee that the insurance company can expect to pay out at a rate that closely mimics the average incidence of risk in the population, about which the insurance company is presumed to have reliable knowledge. This last requirement may not obtain in a country context like India's, where the regulator is sitting on an aggregated mass of claims information that is not shared widely with insurers.

While risk pooling would keep total losses predictable, it is still possible for the insurer to experience greater claims than the premiums collected during any particular period. To account for such a scenario, insurers must maintain reserves that can be used to cover unexpected losses. Such reserves can be funded through insurers' own equity, or raised in capital markets at a cost, but the presence of well-functioning capital markets is required for such costs to be manageable. Again, this latter condition may fail to obtain in developing countries.

The market structure also matters. An oligopolistic form may allow for better pooling possibilities, but it can also collude to make entry difficult for newer and likely more efficient suppliers. Moreover, the regulator may erect entry barriers in the form of high capital requirements or price controls, thereby precluding optimal entry and exit rates on the supply-side.

### Contract features

The second set of issues arises concerning the contractual arrangement between the insurer and the insured. Here, information asymmetries and the associated problems of adverse selection and moral hazard are key points of concern, as the insurers' rational response to these problems will dictate what and how much they will cover, or the benefits package, and the prices they will charge, or the loading. There are other features of the contract also that deserve consideration, but here we focus on the two most important features.

#### Benefits package

Setting the benefits package involves deciding the type of services to be financed and the number of benefits provided (2, 26). An insurer will have to consider the implications of information asymmetries involved when deciding the scope of the benefits package. Inclusion of services with high price elasticity of demand, for instance, could result in unnecessary use of care, leading to higher claim payouts with hardly any additional benefits to the insured. In addition to information asymmetries, an insurer must consider a balance of multiple factors when designing the benefits package. For instance, a broader benefits package allows insurers/investors to diversify their risks better. Furthermore, where there is synergy among existing benefits, insurers are likely to benefit from the economies of scope in production, distribution and marketing they allow. In all of this, the insurers also have to consider what the consumers want if they hope for it to be taken up (26). The design of benefit packages, in the short term is often influenced by information asymmetry concerns of insurers. However, in the long run, for insurance to actually result in the dual objectives of more effective financial protection and better health outcomes for customers, insurers will have to consider comprehensiveness as a key principle in benefit design.

#### Loading

The premium that an individual has to pay in order to be insured is not limited to not just the “actuarially fair” premium (to cover the claims incurred by the insurer during the year), but also other administrative costs associated with the product (for selling, claims processing, etc.). It is this component-loading, that is charged in addition to the net premium that decides cost recovery and expected profits of insurers (26). As with the benefits package, a decision regarding the loading costs charged also has to be taken considering a set of factors, including the impact of information asymmetries at play. For instance, moral hazard, both ex-ante in the form of the probability of illness, influenced by the preventive efforts of the insured as well as ex-post, where the insured demands more or higher-cost care, has an impact on loading (7). An expectation of either or both can cause insurers to increase loading. These increased payouts also come with a greater administrative cost burden owing to claims management, further increasing loading. Similarly, the anticipation of adverse selection (which could be higher when risk-averse insurance managers decide on behalf of investors) could result in insurers viewing customers riskier than they are, leading to a higher loading factor to be charged.

When insurers acquire reserves, there is an additional cost of capital involved to enable the insurer to pay out unexpectedly high claims. Loading, then, also includes the transaction costs associated with acquiring these reserves.

Loading also becomes higher when the costs involved in acquiring customers is high. For instance, as discussed earlier, not all individuals may be aware of insurance or comprehend the risks posed by sudden health expenditures on their finances. In such cases, the costs involved in acquiring customers also add to loading. However, these acquisition costs also positively impact, leading to a potential increase in the pool size. As the size of the pool increases, losses become more predictable, and as a result, the reserves needed per unit risk to attain solvency also goes down, reducing loading. In addition, the delivery channels used to get the products to the insured and the costs associated with each can impact loading. Also, the benefits package itself can have a direct bearing on costs. The broader the benefits package, the more likely it will trigger moral hazard and hence a need for higher loading.

There are, thus, several factors that may push loading to higher levels. The problem here is that this could lead to a situation where the benefits offered may not be worth the resultant high premium triggering a host of issues, including a reduction in demand itself. Hence the contract must carefully choose the right balance of benefits package and premium.

### Purchaser provider relation

When it comes to health insurance, healthcare service providers are key players to account for. While the insurance contract allows insurers to pay for care the insured needs, thereby financially protecting them, this arrangement does not provide insurers with a sense of the quality-of-care the insured gets and its impact on their health outcomes. There is yet another information asymmetry at play here, with the providers being the technical experts in healthcare matters with information not easily accessible to either consumers or insurers paying on their behalf. This information asymmetry in which an indemnity insurance contract operates can give providers an incentive to maximize profits by prescribing more treatments or costlier treatments with little to no benefit to consumers.

To have better control over the cost and quality of care and, with it, the health outcomes of the insured, insurers would need to have a more active relationship with the provider. This would require insurers to build a better understanding of the factors influencing the decisions of providers. One way in which insurers can actively engage with the provider space is through strategically structuring provider payment. Instead of passively reimbursing medical expenditure incurred, insurers can tie providers' incentives to quality-of-care or health outcomes. For instance, Geynor et al. note that when a patient choice is not responsive to price (as in the case of insured), “greater competition will lead hospitals to optimally increase their quality” (25). Providers with higher quality then have the scope to bargain with insurers for higher prices.

Thus, the kind of relationship insurers have with providers has important implications on the final output consumers receive and the long-term costs facing them. This relationship can take different forms, with insurers exercising varying levels of control over the cost and quality of care based on how integrated they are with providers (26). This degree of vertical control is in turn determined by the relative market power of insurers and providers, management know-how for insurers, insurers' ability to raise capital, among others.

While in this paper we do not examine the factors affecting a provider's objective, behavior or business decisions, there is an evident need to study these in greater detail to fully understand how insurer's interaction with providers can be framed.

## Getting the right alignment of incentives

When one side of a contract knows more than the other, it can cause the former to exploit this advantage. These asymmetries in information, impact both the demand and supply of insurance, distorting the market. While we have briefly touched upon some of the barriers posed by information asymmetries, we believe they merit a more focused discussion to examine the different forms they take and how they impact demand and supply.

### Consumer has information that insurer does not

In the case of insurance, the seller cannot observe the consumer's probability of facing a health shock, and this lack of information can lead to people with higher risk choosing to hedge the risk, preferably without paying more for the greater risk (*adverse selection*) (27). This leads insurers to assume that everyone who chooses to buy insurance is a bad risk. This then pushes insurers to charge higher premiums to cover for the potentially high payouts. What this, in turn, does is to keep out low-risk individuals as the premium they now have to pay is more than their perceived risk. Adverse selection thus can lead to a lower number of policies sold in the market, prevent the existence of a stable market, and lead to the market disappearing (8).

The second kind of issue that stems from consumers having greater information than insurers is associated with the impact insurance may have on the amount of loss itself. Insurance can sometimes impact the loss itself (*moral hazard*) (8). This can take two forms: (1) The provider is unable to observe the steps the consumer is taking toward maintaining his health, making it difficult to discern their type. With the provision of insurance, the cost of a health shock is reduced considerably, and with it, the incentives that consumers have, to maintain health. (2) As insurance reduces the user price of medical care, consumers have incentives to demand more and possibly costlier care (28). This may not necessarily be a bad thing as it could improve welfare in a developing country context such as India's, with their low medical care availability and utilization. However, uncontrolled and unnecessary use of care service can eventually lead to medical cost inflation without any significant improvement in quality (21).

### Health care providers have more information than consumers/insurers

Another manifestation of information asymmetry is in the relationship between care providers and consumers, where the latter lack the information the former may have. When it comes to treatment decisions, consumers rely almost entirely on the provider's judgement owing to the higher technical knowledge the latter holds. Here insurers can act as expert agents of the consumer. However, with little to no power over providers, they do little else from reimbursing providers for the treatment provided. Reimbursement rates under insurance, as seen earlier, can then create incentives for providers to provide either more services than necessary or more expensive treatment options which may hardly benefit consumers (5). The rising prescription of hysterectomies to women, particularly higher among private providers, is an example. These surgeries are highly profitable for providers and often are prescribed without providing less permanent choices to women (29).

Additionally, Sengupta and Rooj note that providers tend to inflate treatment fees for insured patients (21). Through overprovision of services, overbilling of services and prescription of costly procedures, providers could induce moral hazard, as noted earlier. An indemnity insurance contract, then, with limited control from the payer, leaves space for providers to overcharge, eventually inflating health spending systemwide. Thus, the nature of the financing arrangement, and by implication, the form and substance of provider-insurer coordination or integration, is a major factor driving health spending inflation, as Hsiao also surmises (30).

### Insurers have more information than consumers

Compared to consumers, insurers enjoy an advantage when it comes to knowledge of the insurance product itself. Owing to the complexity of insurance policies, consumers are often left confused. While some of this may result in a non-purchase of insurance, consumers with sufficiently high-risk aversion may still choose to purchase, despite this lack of clarity about the product. Insurers can exploit this information asymmetry to sell unsuitable products. In this case, it is highly likely that the consumer's expectation of the product is quite different from the product itself. This could be exacerbated when there is an agent involved acting as an intermediary who would then have his/her own incentives to promote policies for which he/she would receive higher commission over, say, more consumer-friendly policies (31).

Here, the consumer need not necessarily be an individual client, but could also be employers or the government acting on behalf of a larger group of people. Despite the collective bargaining power, given the opacity of the insurance market, they may still have to rely on insurers for advice on suitable products, leaving scope for insurers to exploit the information asymmetry here.

## Discussion: In search of a solution

It is clear from the discussion above that, multiple distortions make the current indemnity-based voluntary commercial health insurance an unlikely pathway to achieve the twin UHC objectives of improved health outcomes and financial protection. But commercial health insurance still offers potential to develop solutions to For commercial health insurance in India to become better aligned with and support the path to UHC, there is a clear need to reimagine the space as we know it now. For a functioning insurance market that is available to all, a market must be designed that minimizes actual demand and supply distortions.

One way to reimagine commercial health insurance would be through an integration of systems of care and insurance. Such a model is expected to align incentives and lead to efficiencies. With this alignment of incentives, we envision costs of care to go down and quality, owing to internal accountability measures arising from integration, to improve. Incentive alignment is thus expected to lead to better health and financial protection of individuals. A demonstrated impact can then help build the intertemporal trust that is crucial for sustained demand for insurance in a repeated game.

One element of this integrated model would be an active gatekeeping function carried out at the primary level of care. This is then expected to reduce moral hazard and mitigate some of the effects of adverse selection. With keeping people healthy than reimbursing when sick being the operative feature of such a system, an integrated model is also expected to be more proactive. Perceivable benefits that such a proactive system can offer (in the form of primary care consultations, regular screening, etc.), we expect, would also reduce demand-side barriers like loss aversion. It also keeps members better aware of their health status. Given that the system's responsibility is the health of its members, which goes beyond simply dealing with illness, the system is expected to work around hyperbolic discounting in care-seeking.

A single large such integrated player providing care and insurance would hold the most power and have the maximum number of the service provider at its disposal. However, such a system has also been seen to come with a set of inefficiencies in the form of long wait times, reduced innovation, etc. (32). This is where competing systems are expected to have an advantage. In a competing model of integrated systems, consumers or expert agents acting on behalf of them (could be the government, employer, etc.) will reward with more subscribers those plans that provide the most value for money in terms of: improved quality, reduced cost and higher customer satisfaction (33). Israel's competing health plans offer an interesting example to study in this regard.

Even when complete integration is not possible, the benefits of the system may be largely replicable where insurers contract with a network of primary care providers. This relationship can take the form of using pooled money to pay primary care networks (34) or direct acquisition of primary care providers for care provision (35). Such a focus on primary care sets a clear path to reducing risks of future hospitalization. The savings that accrue to insurers from the lowered-risk profile, owing to primary care, are shared back with primary care providers. This additionally adds value to the customer offering them more visible benefits as opposed to reimbursements post hospitalization.

Insurance products currently in place are quite complex in nature. Price and quality comparisons are quite difficult to make, leading to high search costs. In the presence of high costs, individuals may decide that the benefits are not worth the cost and choose not to buy insurance. Facilitating easy comparison of insurance products can have a positive impact on demand. This can take the form of standard products for comparison, insurance exchanges, etc.

No solution-design is going to be complete without exploring the role of regulators. Regulatory frameworks should accompany the design of structures and processes to minimize distortions so that the solution does not end up resulting in exacerbating the problem itself. Regulatory enablers such as data portability, quality and transparency standards, and floor-level requirements on insurers/providers for redressing consumer grievances can have a critical role to play in achieving the twin objectives.

## Data availability statement

The original contributions presented in the study are included in the article/supplementary material, further inquiries can be directed to the corresponding author.

## Author contributions

HA and IG wrote the first draft of the manuscript. All authors contributed to conception the study paper, contributed to manuscript revision, read, and approved the submitted version.

## Conflict of interest

The authors declare that the research was conducted in the absence of any commercial or financial relationships that could be construed as a potential conflict of interest.

## Publisher's note

All claims expressed in this article are solely those of the authors and do not necessarily represent those of their affiliated organizations, or those of the publisher, the editors and the reviewers. Any product that may be evaluated in this article, or claim that may be made by its manufacturer, is not guaranteed or endorsed by the publisher.
